# Regulation of Ion Transport in the Intestine by Free Fatty Acid Receptor 2 and 3: Possible Involvement of the Diffuse Chemosensory System

**DOI:** 10.3390/ijms19030735

**Published:** 2018-03-05

**Authors:** Atsukazu Kuwahara, Yuko Kuwahara, Toshio Inui, Yoshinori Marunaka

**Affiliations:** 1 Division of Molecular Cell Physiology, Kyoto prefectural University of Medicine, 465 Kajii-cho Kamigyo-ku, Kyoto 602-8566, Japan; kuwaya28@koto.kpu-m.ac.jp (Y.K.); marunaka@koto.kpu-m.ac.jp (Y.M.); 2 Saisei Mirai medical corporation, 6-14-17 Kinda, Moriguchi, Osaka 570-0011, Japan; t-inui@saisei-mirai.or.jp

**Keywords:** diffuse chemosensory system (DCS), short-chain fatty acid (SCFA), free fatty acid receptor 2 (FFA2), free fatty acid receptor 3 (FFA3), enteroendocrine cell (EEC), intestine, chloride secretion, bicarbonate secretion

## Abstract

The diffuse chemosensory system (DCS) is well developed in the apparatuses of endodermal origin like gastrointestinal (GI) tract. The primary function of the GI tract is the extraction of nutrients from the diet. Therefore, the GI tract must possess an efficient surveillance system that continuously monitors the luminal contents for beneficial or harmful compounds. Recent studies have shown that specialized cells in the intestinal lining can sense changes in the luminal content. The chemosensory cells in the GI tract belong to the DCS which consists of enteroendocrine and related cells. These cells initiate various important local and remote reflexes. Although neural and hormonal involvements in ion transport in the GI tract are well documented, involvement of the DCS in the regulation of intestinal ion transport is much less understood. Since activation of luminal chemosensory receptors is a primary signal that elicits changes in intestinal ion transport and motility and failure of the system causes dysfunctions in host homeostasis, as well as functional GI disorders, study of the regulation of GI function by the DCS has become increasingly important. This review discusses the role of the DCS in epithelial ion transport, with particular emphasis on the involvement of free fatty acid receptor 2 (FFA2) and free fatty acid receptor 3 (FFA3).

## 1. Introduction

The primary function of the gastrointestinal (GI) tract is obtaining energy sources from the diet as nutrients. The intestinal lumen is open to the external environment, and the GI tract is a one-way tube in which food materials are progressively converted into molecular end products. Furthermore, approximately 100 trillion gut microbiota are present in the colonic lumen [1]. The gut microbiota make a diverse bacterial community that is separated from the internal environment by a single layer of epithelial cells. In addition, the gut microbiota continuously produces a large amount of various chemicals including short-chain fatty acids (SCFAs) using the microbiota genome which has been estimated to contain 150-fold more genes than the host genome [1]. Among them, some are beneficial and others are harmful to the host. Thus, host–microbiota interaction occurs along mucosal surfaces in the intestine of the host and, in the gut, host-defense mechanisms operate to maintain a healthy gut. One such mechanism is the ability to flush out harmful substances via fluid secretion, mainly Cl^−^ secretion. In addition to such a defense mechanism, mild fluid secretion is also important to provide appropriate luminal fluidity for commensal bacteria and flush out the harmful substances derived from bacteria and other toxins to maintain a niche of commensal bacteria. Furthermore, Cl^−^ secretion significantly influences the composition of the commensal bacteria inhabiting the GI tract [2]. Large quantities of water and electrolytes are also secreted or absorbed to achieve the digestive process. Intestinal fluid secretion is initiated by various reflexes through chemosensory receptors, ion channels or transporters located on intestinal epithelia in addition to nervous and humoral influences. 

Until recently, the essential role of taste was considered to be the detection of nutrients and poisonous substances in the tongue. However, the taste receptors including several taste signaling molecules have been demonstrated to be expressed in many extraoral locations (e.g., GI tract, respiratory system, heart, brain, kidney, urinary bladder, adipose tissue, testis, spermatozoa, lymphocytes, and endocrine glands) [3,4,5,6,7,8]. This wide distribution suggests that taste receptors may have functional roles far beyond the original concept of taste perception.

In the GI tract, nutrients and microbial metabolites are continuously sensed by chemosensory receptors which are analogs of taste receptors in the tongue [9,10,11]. Although these chemosensory receptors in the stomach and intestine do not trigger taste sensations, such chemical detection elicits appropriate metabolic responses in the host. The chemosensory receptors are usually expressed on epithelial cells. Chemosensory receptors expressing cells in the GI tract belong to a part of the diffuse chemosensory system (DCS) [12]. In the GI tract, DCS consists of enteroendocrine and brush cells [13,14]. These cells initiate various important local and remote reflexes. Despite their obvious physiological and clinical importance, not enough information is available regarding the function of DCS in the GI tract. 

In this review, we summarize our current knowledge of DCS in the GI tract and discuss possible involvement of this system in the regulation of electrolyte transport stimulated by gut microbiota metabolites, SCFAs through FFA2 and FFA3 activation. In Section 2, Section 3 and Section 4, we describe general information of DCS for the reader’s convenience. Then, we discuss the possible involvement of DCS in ion transport stimulated by FFA2 and FFA3 based on our original studies. 

## 2. Diffuse Chemosensory System

In the apparatuses of endodermic origin (i.e., the GI and respiratory tract), the DCS is composed of solitary chemosensory cells (SCCs, also called solitary chemoreceptor cells). They have analogies to taste receptor cells but are not aggregated in buds [15,16]. First evidence for chemosensory function of these cells in DCS arose from the immunohistochemical detection of a key molecule, α-gustducin, a primarily taste-specific G protein α-subunit, in these cells and after that it became clear that SCCs detect bacterial products via the bitter taste signaling cascade to induce protective local and systemic reflexes in the respiratory tract such as reduction of respiratory rate, local neurogenic inflammation and secretion of antimicrobial peptides [17,18,19,20,21,22]. α-gustducin was also detected in the gut epithelial cells by Hӧfer et al. [23], raising the possibility that cells outside the oral cavity may use taste receptors as chemosensory receptors. In other words, the DCS may have crucial physiological roles in the GI tract. These cells are now interpreted as sentinels monitoring the mucosal surface for the presence of potentially hazardous compounds or beneficial compounds for our body. 

## 3. Chemosensory Cells in the Intestine

### 3.1. Enteroendocrine Cell

Enteroendocrine cells (EECs) scattered along the epithelial layer of the GI tract from the stomach to the rectum are considered to be chemosensory cells of the DCS [14]. EECs comprise 0.1–1% of the gut epithelium and produce gut hormones or peptides that orchestrate the body’s response to food ingestion. Most EECs are polarized, with long microvilli at the apical surface projecting into the intestinal lumen. Granules containing hormones are concentrated around the basolateral side of the cells. EECs are divided into at least 15 different types depending on the hormone they produce [14] (Table 1). Enterochromaffin cells (ECs) are a type of EEC that resides in the gut epithelia. ECs secrete 5-hydroxytryptamine (5-HT) and released 5-HT regulates secretory and motility reflexes. As EECs directly face into the intestinal lumen, they are ideally placed to detect luminal chemical compounds including the composition of ingested food and bacterial metabolites. Thus, EECs function as primary sensors of ingested nutrients or chemical compounds existing in the gut lumen. A large number of EECs respond to luminal chemical compounds by secreting a variety of gut hormones. Released gut hormones from EECs play important roles in physiological functions including glucose metabolism, feeding behavior or energy balance, etc., and is the first step in the gut–brain axis [24,25]. The secretion of peptides and hormones from EECs is mainly triggered by the sensing of luminal contents via G-protein coupled receptors (GPCRs), including FFAs. Recent gene expression profiling studies suggest that EEC characteristics are based on a combination of GPCRs that vary among GI segments [26]. Furthermore, there is a high degree of transcriptional overlap between different EEC cell populations [26] and it is not clear whether the individual cells containing hormones are determined predominantly by their location along the GI axis, or whether they are also significantly influenced by ingested foods and metabolites. Since the immunoreactivity of many nutrient GPCRs is often detected in the cytosol, it is not clear which side (apical or basolateral) of the epithelial cells express nutrient GPCRs. Indeed, a recent study has shown that Glucagon-like peptide 1 (GLP-1) secretion stimulated by FFA1 agonists appears to require absorption prior to stimulating GLP-1 secretion using an isolated, perfused small intestine from a rat model [27]. This finding suggests that nutrient absorption, in some occasions, is required for nutrient GPCR activation to stimulate gut hormone release. 

### 3.2. Brush Cells

Other candidates for chemosensory cells in the GI tract are brush cells (also termed as peculiar, tuft, multivascular, caveolated, fibrillovesicular, S, or agranular light cells) [28]. It was originally reported that cells immunoreactive for gustducin located in the rat stomach were brush cells [23]. Brush cells express T1Rs, the Ca^2+^-gated monovalent cation channel transient receptor potential subfamily of M (TRPM5) and gustducin, all of which are molecules that are important for taste transduction. Brush cells are pear- or bottle-shaped with a broad perinuclear portion located at the base. An apical microvillus tuft is in contact with the intestinal lumen. Although brush cells occur with a high frequency in the cardia of rat stomach, in the rat common bile duct, and in the pancreatic excretory duct, brush cells are also present throughout the GI tract including salivary and bile ducts and contain various bioactive products [28]. Therefore, these cells may imply a role in the detection and/or regulation of intraluminal pressure. However, brush cells lack chromogranin immunoreactive granules, thus, they may synthesize transmitter chemicals de novo, releasing them predominantly into the lumen or submucosal space. Differentiation into brush cells depends on the transcription factor atonal homolog 1, but does not require neurogenin 3, which is essential for EEC differentiation, indicating that brush cells are distinct from EECs [29]. 

## 4. FFA Receptors in the Intestine

Free fatty acids are essential nutrients affecting various cellular function. Free fatty acids compose of carboxylic acid linked to an aliphatic tail of varying chain length. Therefore, free fatty acids are generally classified and defined on the basis of their aliphatic chain. Free fatty acids exert their biological effects through several signaling pathways, but the precise mechanisms are still unclear. 

SCFAs are the predominant anions in the content of the large intestine, existing at a concentration of ~100 mM, and mainly consisting of acetate, propionate, and butyrate. They are produced by bacterial fermentation of specific indigestible dietary fibers and oligosaccharides. Variation in the composition of gut microbiota responsible for SCFA production is now known to be frequently linked to health and disease [30,31,32] and many studies are demonstrating that SCFAs are the key signaling molecules underlying this link.

It is known that GPCRs are the largest family of transmembrane signaling polypeptides encoded within genomes of eukaryotic species [33]. It is now recognized that several GPCRs are activated by various free fatty acids. Free fatty acid recognition via GPCRs is dependent on carbon number [34]. This includes four receptors that have been officially classified as members of an FFA family: FFA1–FFA4 [35]. Two of these receptors, FFA1 (previously designated as GPR40) and FFA4 (previously GPR120) are activated by middle-chain fatty acids and long-chain fatty acids, while the remaining two, FFA2 (previously GPR43) and FFA3 (previously GPR41) are activated by SCFAs. In 2003, FFA2 and FFA3 were deorphanized as SCFA receptors [36,37]. FFA2 and FFA3 are structurally related and share ~40% amino acid sequence similarity. These receptors are encoded in tandem on chromosome 19 in humans [38] and are similar across several mammalian species. They differ in affinity for SCFAs, tissue distribution, and physiological roles. FFA2 has similar affinity for acetate, propionate and butyrate, but FFA3 has different potency order; propionate > butyrate >> acetate. Thus, acetate preferentially activates FFA2, propionate activates FFA3, and butyrate preferentially activates FFA2 and FFA3. FFA2 and FFA3 have distinct G protein-coupled in their intracellular signaling cascades; FFA2 couples both pertussis toxin-sensitive (G_i/o_) and -insensitive (G_q_) G protein and FFA3 only to G_i/o_ protein [36,37,39]. 

Since SCFAs are mainly produced by bacterial fermentation of indigestible dietary fiber in the colon, it was predicted that FFAs were expressed in colonic epithelial cells. Indeed, we have demonstrated that FFA2- and FFA3-immunoreactivity was found in rat, guinea-pig and human colonic epithelia, with particularly strong expression in PYY and GLP-1-producing EECs (L cells) but not 5-HT containing EC cells [40,41,42,43]. Immunoreactivity for FFA2 in laboratory animals showed a similar pattern to those in the human colon. FFA2-immunoreactive L cells in the colon were open type extending their cell body to the luminal surface. FFA3 was also detected in human colonic open type L cells, but it is still unclear whether these two receptors are located in the same cells. These morphological studies indicate that PYY- and GLP-1-containing L cells expressing FFA2 and FFA3 are chemosensory cells and activation of these receptors by luminal SCFAs may trigger PYY and/or GLP-1 secretion. From a morphological point of view, it is still unclear whether FFA2 or FFA3 are confined to apical or basolateral membrane. FFA3 is also expressed in the autonomic, somatic sensory and enteric nervous system (ENS) [44,45]. In addition, both FFA2 and FFA3 are found in various immune cells including monocytes and polymorphonuclear cells [36,37,40].

In addition to the current FFA family members, there are several additional GPCRs reported to be activated by SCFAs; mouse olfactory receptor Olfr78 (OR51E2 in human) appears to be activated by the SCFAs [46].

## 5. Involvement of FFA2 and FFA3 on Intestinal Ion Transport

### 5.1. Duodenum

The duodenal epithelium is periodically exposed to strong gastric acid combined with bicarbonate secreted from the duodenal epithelial cells and from the pancreas. This cyclic change in luminal pH is more pronounced postprandially and varies the luminal pH between two and seven in a time scale of minutes [47,48,49]. Rapid shifts in duodenal pH are likely to create intense stress on the duodenal epithelial cells to maintain constant intracellular pH to preserve function and prevent irreversible necrosis as a result of intracellular acidification [50,51]. Thus, a potent defense mechanism must be in place to prevent cellular acidification during intraluminal pH changes. 

Intestinal SCFA sensing and uptake have been studied primarily in the colon due to a large amount of SCFA in the colon [52,53]. However, SCFAs are also present in the duodenal lumen at 0.1–1 mM, derived from the fermentation of nutrients by oral flora and from condiments, and fermented and preserved foods [47]. For example, acetic acid is frequently ingested in the diet since the common condiment vinegar is 4–7% acetic acid by volume (~0.7–1.2 M). In addition, oral flora is another source of acetate [47]. Thus, the duodenal mucosa may be exposed to concentrations of SCFAs sufficient to activate cognate sensors such as FFA2 and FFA3. 

Immunoreactivity of FFA2 and FFA3 was detected in cells of endocrine-like morphology in the duodenal mucosa [54]. FFA2 colocalized with 5-HT-cointaining ECs and FFA3 colocalized with GLP-1-containing L cells. These results indicate that an expression pattern is different from FFA2 colocalization in the colon, and consistent with segmental differences in endocrine cell expression. In the duodenum, a 35-kDa band was also present in duodenal mucosa close to the predicted size of the rat FFA2 (37 Kd) [54].

The most studied duodenal defense mechanism is epithelial bicarbonate (HCO_3_^−^) secretion. Bicarbonate secretion is considered to neutralize luminal acid in the pre-epithelial mucus gel, preventing acid contact with epithelial cells [55,56].

In the duodenum, FFA2 and 3 differentially modulate duodenal HCO_3_^−^ secretion stimulated by SCFAs [54]. Luminal perfusion of acetate dose-dependently increased HCO_3_^−^ secretion and the secretion was increased by prior injection of the Dipeptidyl Peptidase-4 (DPPIV) inhibitor, 6-[2[2-(2-cyanopyrrolidin-1-yl)-2-oxoethyl]ethylamino]pyridene-3-carbonitrile dihydrochloride (NVP728) luminal perfusion of propionate (0.1 mM) also increased HCO_3_^−^ secretion and this was enhanced by NVP728. Interestingly, acetate-induced HCO_3_^−^ secretion was partially inhibited by the monocarboxylate transporter 1 (MCT1) inhibitor, 4-CHCA. These data suggest that luminal SCFAs increase HCO_3_^−^ secretion in part via SCFA transport via MCT1.

**Figure 1:**
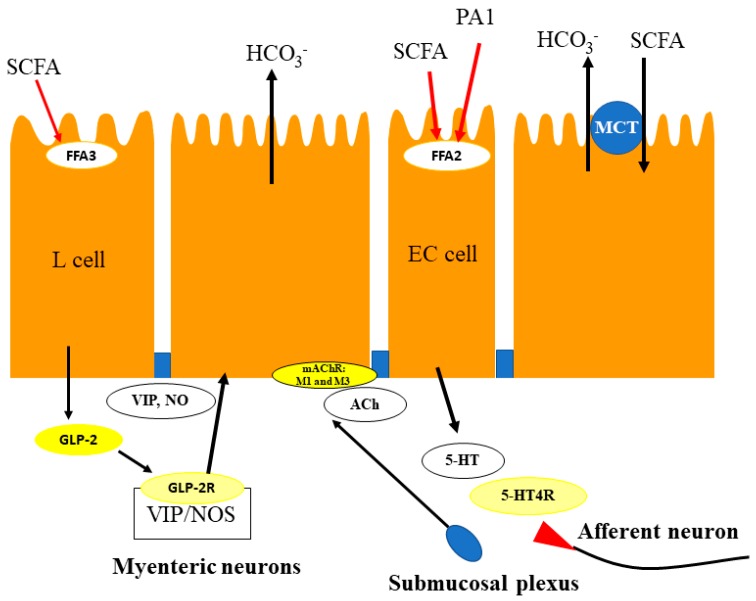
Proposed mechanism of duodenal SCFA sensing. Luminal SCFAs, derived from diet and oral microbiota activate multiple pathways to induce HCO_3_^−^ secretion in duodenum. SCFAs may activate FFA3 on L cells to release GLP-2. Released GLP-2 activates GLP-2 receptors located on endothelial nitric oxide synthease (e-NOS)-expressing and VIP-positive myenteric neurons followed by the release of VIP and NO. Then, release VIP and NO stimulate epithelial HCO_3_^−^ secretion. SCFAs also activate FFA2 on EC cells, which release 5-HT and ACh, activating 5-HT_4_ and muscarinic receptors, respectively, with both expressed on enteric and on epithelial cells to stimulate epithelial HCO_3_^−^ secretion. SCFAs might be absorbed by apical membrane MCT1, increasing the rate of HCO_3_^−^ secretion. SCFA, short-chain fatty acid; GLP-2, glucagon-like peptide 2; VIP, vasoactive polypeptide; NO, nitric oxide; 5-HT, 5-hydroxytryptamine; ACh, acetylcholine; EC, enterochromaffin; MCT, monocarboxylate transporter. PA-1, phenylacetamide-1.

Luminal perfusion of the selective FFA2 agonist, PA1 stimulates duodenal HCO_3_^−^ secretion and the response was affected by prior injection of atropine but not by NVP728. In addition, muscarinic receptor 1 (M1) antagonist, telenzipine or M3 antagonist J104129 reduced PA1-induced HCO_3_^−^ secretion. Furthermore, the 5-HT_4_ receptor antagonist GR113808 abolished PA1-induced HCO_3_^−^ secretion but the 5-HT_3_ antagonist, ondansetron had no effect. Therefore, FFA2 activation-induced HCO_3_^−^ secretion is linked with muscarinic and 5-HT_4_ receptor activation (Figure 1). The results also indicate that FFA2 is functionally expressed in EC cells and that FFA2 activation releases 5-HT, followed by ACh release and not by peptide release. FFA2/3 mixed agonist, acetate-induced HCO_3_^−^ secretion is reduced by combination of GR113808 and GLP-2 receptor antagonist, GLP-2(3-33), but each antagonist alone had little effect indicating that luminal SCFAs activate both FFA2 and FFA3, followed by 5-HT and peptide release, respectively. Luminal perfusion of acetate increased GLP-2 concentration of portal vein and enhanced further by NVP injection [54]. Acetate and propionate are reported to increase GLP-1 release by activation of FFA2 from primary colonic cultures [57], whereas PA1 had no effect on GLP-2 release in the duodenum. Therefore, acetate-induced increase in HCO_3_^−^ secretion may be involved in the activation of FFA3 via GLP-2 dependent and independent pathways (Figure 1). GLP-2-induced HCO_3_^−^ secretion is supported by following similar experiments that luminal perfusion of l-glutamate and 5′-inosine monophosphate increases duodenal HCO_3_^−^ secretion via GLP-2 release and GLP-2 receptor activation, followed by nitric oxide and VIP release [58,59]. 

Bicarbonate secretion stimulated by FFA2 and FFA3 in the duodenum is considered to a defense mechanism because duodenal mucosa is regularly exposed to gastric acid, bile acids and nutrients. Therefore, luminal chemical sensing in the duodenum is needed to neutralize acids in the duodenal lumen by HCO_3_^−^ secretion. Because the pKa of SCFA is ~5, SCFAs are mostly dissociated at neutral pH, existing in the anionic form, which may be absorbed via transporters such as MCT, rather than what has been historically proposed, i.e., absorbed passively in the undissociated form through the colonic epithelium at luminal pH < 6 [60,61]. Therefore, the stimulation of HCO_3_^−^ secretion by luminal SCFA may help the absorption of SCFA anions in the duodenal mucosa by raising the luminal pH, and thus, increasing the relative concentration of the anion form.

In conclusion, duodenal EECs express functional FFAs; FFA2 colocalizes with 5-HT and FFA3 colocalizes with GLP-1. FFA3 activated by SCFA presumably increases HCO_3_^−^ secretion via release of GLP-2, whereas FFA2 activation induces HCO_3_^−^ secretion via muscarinic and 5-HT_4_ receptor activation. With respect to physiological significance, SCFA-induced HCO_3_^−^ secretion in the duodenum is a considered novel pathway to locally regulate hormone and mediator release, and may serve important mechanisms for mucosal protection and repair.

### 5.2. Proximal Colon

Proximal colon is always exposed to high concentration of SCFAs, which is produced by microbial fermentation in the caecum, where they are used locally by enterocytes or transported across the colonic epithelia into the bloodstream. In addition, SCFAs in the lumen functions as chemical signaling molecules through the activation of FFAs. Changes in the gut microbial population or composition ratio is well known to be implicated in the pathogenesis of GI or metabolic disorders [62,63]. Therefore, to monitor composition and population of luminal SCFAs is important for luminal microbial fermentative activity. 

Segmental heterogeneity of electrolyte transport in the colon has been previously observed in humans and other species [64,65,66]. For example, propionate and butyrate but not acetate, induce Cl^−^ secretion in the rectum, as well as in the distal and middle colon. On the other hand, propionate and butyrate do not stimulate Cl^−^ secretion in proximal colon. Furthermore, enteric neurons are reported to affect electrolyte transport activity in the proximal colon; activation of submucosal neurons led to release of ACh and other neurotransmitters and an increase in short-circuit current (Isc) that reflect inhibition of Na^+^ and Cl^−^ absorption [67]. Since FFAs, especially FFA3 are ubiquitously expressed in autonomic and sensory neurons [44,68,69], neuronal activation of FFA3 might be involved in the regulation of electrolyte transport in the proximal colon. Indeed, Kaji et al. recently reported that FFA3 contributes to epithelial secretory action through suppression of enteric neuronal activity in the proximal colon of rats [45]. 

FFA3 immunoreactivity was detected in the myenteric and submucosal plexuses located in the nerve fibers and endings rather than in the neuronal cell bodies in addition to EECs in the rat proximal colon [45]. FFA3-positive nerve fibers surrounded crypts and most of the FFA3-positive nerves coexpressed cholinergic neuronal markers, vesicular acetylcholine transporter (VAChT) and high-affinity choline transporter-1 (CHT1). However, FFA3 did not express VIP, calbindin or α-CGRP-positive neurons which innervated the colonic wall. Thus, FFA3-positive cholinergic nerves are separated from the VIP-ergic nerves. There are two major secretomotor neurons innervating the intestinal epithelium; one is cholinergic secretomotor neurons and another is VIP-ergic neurons but both nerves are closely approximated in rat proximal colon. At present, the origin of FFA3-immunoreactive nerves are unknown, thus, further studies are needed to identify their contribution of SCFAs-induced anion secretion. However, FFA3-positive cholinergic nerve fibers and endings probably are coming from myenteric neurons and/or extrinsic neurons [45,70,71,72]. 

Mucosal and submucosal cholinergic-mediated pathways are important for regulating colonic ion transport [73]. For example, luminal propionate induces Cl^−^ secretory response and circular muscle contractions in rat distal colon through activation of cholinergic neurons in ENS and the responses are reduced by pretreatment of the tissues with TTX [74,75,76]. These results suggest that the contractile and secretory effects of SCFAs are primarily mediated neural cholinergic pathways. On the other hand, inhibitory effects of SCFAs on GI functions have also been reported; repeated luminal perfusion of SCFAs inhibit colonic motility and fluid secretion via PYY release in rat colon; SCFAs reduce neutrally evoked muscle contractions of isolated rat colon, which are independent of the presence of mucosa [77,78]. Furthermore, we have previously reported that propionate-induced circular muscle contractions were not observed in mucosa-free preparations [75]. These results raise a possibility that SCFAs reciprocally regulate muscle contraction or fluid secretion through enteric neural activity stimulated by mucosal SCFA receptors. 

**Figure 2:**
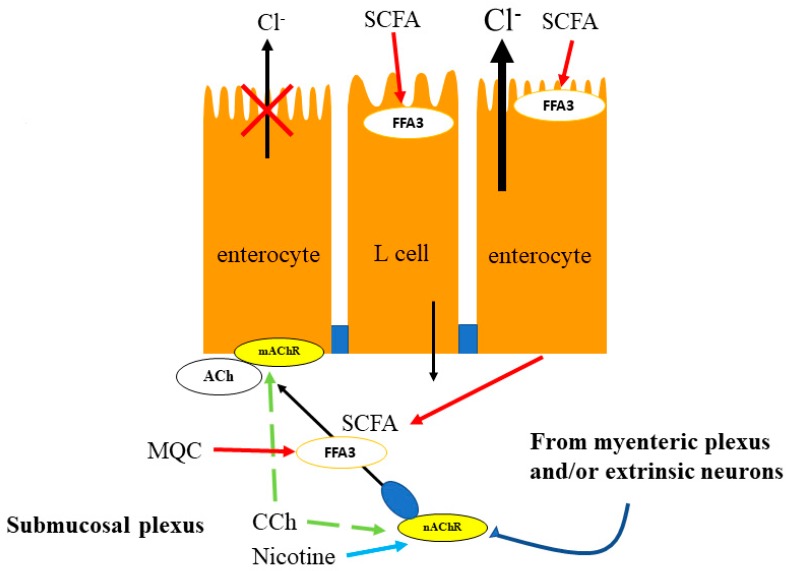
Proposed mechanism of SCFA sensing in the proximal colon. The proximal colonic mucosa is always exposed to high concentrations of microbial metabolite, SCFAs. Non-selective cholinergic agonist, CCh-induced Cl^−^ secretion is decreased by TTX, C6 and the serosal FFA3 agonists, acetate or propionate. Serosal application of a selective FFA3 agonist, MQC also suppresses the response to CCh via the submucosal plexus but not to forskolin. Pretreatment with MQC inhibits nicotine-induced but not bethanechol-induced secretion. The major transmitter of fast excitatory transmission between neurons is ACh, that acts on post synaptic nicotinic receptors [84]. Therefore, cholinergic neuronal axons expressing FFA3 located in the submucosal plexus are possibly activated by CCh and nicotine, and release ACh. MQC or TTX does not affect CCh-evoked secretion in mucosal preparation, where submucosal neurons are eliminated suggesting that MQC has no direct interaction with muscarinic receptors expressed on epithelial cells. SCFA, short-chain fatty acid; TTX, tetrodotoxin; C6, hexamethonium; MQC, *N*-[2-methylphenyl]-[4-furan-3-yl]-2-methyl-5-oxo-1,4,5,6,7,8-hexahydro-quinoline-3-carboxamide; 5-HT, 5-hydroxytryptamine; ACh, acetylcholine; EC, enterochromaffin.

In Ussing chamber experiments, the selective FFA3 agonists *N*-[2-methylphenyl]-[4-furan-3-yl]-2-methyl-5-oxo-1,4,5,6,7,8-hexahydro-quinoline-3-carboxamide (MQC) or AR420626 were demonstrated to reduce cholinergic-mediated Cl^−^ secretion via the submucosal plexus (Figure 2) [45,79]. This anti-secretory effect of MQC is pertussis toxin (PTX)-sensitive, consistent with G_i/o_-coupled FFA3 activation since pretreatment with serosal PTX restored the response to the non-selective cholinergic agonist, carbachol (CCh). Pretreatment of tissues with MQC consistently inhibited nicotine-induced Cl^−^ secretion but did not affect specific muscarinic receptor agonist, bethanechol-induced secretion in mucosa-submucosa preparation. The enteric nervous system contains more than 30 potential neurotransmitters that affect activity [80]. Among them, the major transmitter of fast excitatory transmission between neurons is ACh, which acts on postsynaptic nicotinic receptors [80]. Since atropine abolishes CCh-induced secretion, muscarinic receptors are the end targets of cholinergic-mediated secretion via direct and indirect pathways. In addition, serosal application of propionate or acetate also suppressed CCh-induced Cl^−^ secretion as did MQC. Therefore, cholinergic-mediated Cl^−^ secretion in the proximal colon may be regulated by luminal SCFAs through FFA3 activation in submucosal plexus. Although concentration of SCFAs in subepithelial space is currently unknown, it is probably in the micromolar to millimolar range as a result of the absorption of luminal SCFAs as mentioned below. 

High concentrations (~100 mM) of mixed SCFAs are generated in the cecum by gut microbiota fermentation from indigestible carbohydrates [81]. The uptake of luminal SCFAs in the proximal colon occurred by active cellular uptake of ionized SCFAs via sodium-dependent monocarboxylate transporter 1 (SMCT1) and excreted by MCT1 into the submucosa [82]. A lack of FFA3 was reported to accelerate the intestinal transit rate and decreases the absorption rate of luminal SCFAs [83]. Thus, SCFA sensing in the proximal colon probably reduces motility and secretion, which facilitates fermentation of the luminal content and the absorption of luminal SCFAs. Taken together, neural FFA3 in the proximal colon may be implicated in regulation of the rate of SCFAs absorption via slowing of intestinal transit and inhibition of secretion. From a physiological point of view, suppression of Cl^−^ secretion stimulated by cholinergic secretomotor neurons through the activation of FFA3 is important for the cholinergic reflex that maintains physiological levels of secretion and motility in the proximal colon.

### 5.3. Distal Colon

In the distal colonic epithelia, particularly, PYY and GLP-1 containing L cells in humans, guinea-pigs and rats express FFA2 and FFA3 [40,41,42,43,64]. In contrast to the duodenum, as mentioned above, 5-HT-containing ECs in the colon do not express FFA2 and FFA3 [54]. These results rise a possibility that FFA2 or FFA3-expressed on L cells may have differential contribution for the regulation of SCFAs-induced ion transport in the intestine.

Yajima et al. reported that luminal application of propionate or butyrate but not acetate in the distal colon induced Cl^−^ secretion differed from the proximal colon, where they inversely suppress nicotine-induced Cl^−^ secretion [45,76]. Furthermore, pretreatment of the mucosal surface with procaine or superficial mucosal damage with hypertonic sodium sulfate or xylose inhibits the propionate-induced secretion by 76–90% [74,85]. This observation suggests that propionate-induced Cl^−^ secretion is caused by the activation of SCFA receptors located on mucosal epithelial cells in distal colon. They also reported that neural blockade with TTX and hexamethonium (C6) inhibited the propionate-induced Cl^−^ secretion by 40% and 30% compared with the control, respectively, whereas atropine and local anesthesia with lidocaine remarkably reduce propionate-induced responses by 81–90% and 76–82%, respectively [74,85]. These results indicate that signals from chemosensory cells in epithelial cells traverse a nicotinic ganglion before reaching the efferent neurons that innervate the secretory enterocytes. In addition, propionate-induced Cl^−^ secretion is not affected by tachyphylaxis to capsaicin, CGRP, substance P, histamine or PGE_2_ [85]. From these results, the authors concluded that neither afferent nerves nor arachidonic acid metabolites appear to transmit the signal which releases ACh from secretory nerves, since SP and CGRP are the neurotransmitters associated with afferent nerves [80]. Taken together, SCFA-induced Cl^−^ secretion may be linked to ENS, with involvement of cholinergic secretomotor neurons and non-neural release of ACh but the exact site of ACh release was not fully revealed.

**Figure 3:**
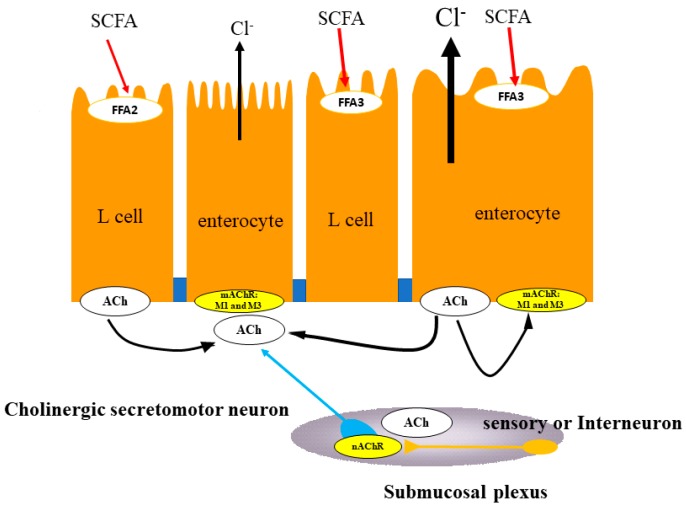
Proposed mechanism of SCFA sensing in the proximal colon. Propionate stimulates chloride secretion via sensory and cholinergic systems of the mucosa in rat distal colon. Luminal addition of propionate and serosal addition of ACh induce Cl^−^ secretion Atropine significantly inhibits propionate-induced Cl^−^ secretion and abolishes ACh-induced response. TTX has no effects on ACh-induced Cl^−^ secretion. Luminal propionate induces ACh release into the serosal fluid. Luminal addition of 3-chloropropionate, an inactive analogue of propionate, abolishes both ACh release and increases in *Isc* response induced by propionate. The non-neural ACh release from distal colonic epithelia coupled with propionate-induced Cl^−^ secretion plays a key role in Cl^−^ secretion, via paracrine fashion of ACh on muscarinic receptors of colonocytes in addition to cholinergic secretomotor neurons.

From the previous studies mentioned above, it was speculated that the remaining 60% of propionate-induced Cl^−^ secretion may be due to the release of ACh from the epithelial cells into the basolateral side because atropine diminished the propionate-evoked Cl^−^ secretion. Indeed, Yajima et al. have recently reported that the luminal addition of propionate induces the ACh release on the serosal side in the mucosal preparations which contain the epithelium and the muscularis mucosae, not including the myenteric and submucosal plexus after concomitant stimulation with propionate-induced Cl^−^/HCO_3_^−^ secretion in the rat distal colon [76]. In the same study, they showed that prior addition of luminal SCFA receptor antagonist, 3-Cl^−^ propionate completely blocked the *Isc* response and abolished ACh release in response to luminal propionate. Furthermore, ACh-induced increases in Cl^−^ secretion in the mucosa preparation are not affected by TTX but is abolished by atropine, suggesting a direct action of ACh on colonic epithelial muscarinic receptors because muscarinic receptors are expressed on intestinal epithelial cells [84]. In rat colonic epithelial cells, M_1_ and M_3_ receptors are reported to be involved in ACh receptor-mediated Cl^−^ secretion [84,86,87]. Therefore, the result suggests that ACh release stimulated by luminal propionate may be from non-neuronal components in the colonic epithelia. Finally, they speculated from their results that ACh-storing epithelial cells have a receptor for propionate, although further studies are necessary to identify specific cells that store ACh (Figure 3). With respect to the involvement of SCFA receptors, FFA3 may be involved in the secretory process since acetate, the preferred ligand of FFA2, has no effect on mucosal Cl^−^ secretion in the distal colon of rats [74]. Unfortunately, the intracellular molecular pathways underlying the effects of SCFAs on colonic Cl^−^ secretion is still not fully understood. Therefore, further study is needed to identify the molecular pathways of FFA-stimulated ion transport in the colon. 

Indigestible dietary fibers are fermented in the cecum and in the proximal colon by anaerobic microbiota, as mentioned previously. Therefore, the proximal colon is continuously exposed to high concentrations of SCFAs but does not secrete Cl^−^ in response to SCFAs because proximal colon actively absorbs SCFAs in combination with a decrease in peristaltic movement to salvage SCFAs as energy sources. On the other hand, the distal colonic mucosa is exposed to SCFAs when semi-solid contents containing SCFAs are transported to the distal colon. Therefore, detection of SCFAs is important in the distal colon as it has the ability to secrete Cl^−^ after SCFA stimulation. In combination with the contractile response, the secretory response to luminal SCFAs in the distal colon seems to function as a lubricant for the movement of luminal contents in the colon. Furthermore, the distal colon and rectum are a boundary between the host and external environment; thus, the high secretory ability of the distal colon is physiologically important for host defense, as it needs to flush out harmful agents in addition to finalizing electrolyte tuning.

## 6. Conclusions

The last six years have seen exciting breakthroughs in the field of chemosensory epithelial cells in the GI tract. As the GI lumen is continuously exposed to various chemical compounds, including nutrients or toxic compounds such as bacterial metabolites and the products of oxidative stress, the chemosensory system in the GI tract is crucial to detect beneficial or harmful substances in the GI lumen. Furthermore, proper fluid secretion in the intestine is important to maintain host homeostasis. 

In this review, we have briefly focused on the relationship between DCS and FFAs in the control of ion transport of duodenum and colon. In summary, contribution of FFA2 and FFA3 in the regulation of ion transport differ among the segment; in the duodenum, EECs express functional FFA2 and FFA3 and both receptors stimulate HCO_3_^−^ secretion through GLP-2 release, muscarinic and 5-HT_4_ receptor activation. In the proximal colon, neural FFA3 activation inversely regulates anion secretion induced by nicotinic ACh receptor activation; in the distal colon, FFA3 activates neural and non-neural ACh release to regulate ion transport. Therefore, we hope that this review stimulates the reader to consider the involvement of the DCS in addition to traditional accepted roles of neural and hormonal factors in the control of intestinal ion transport. To understand the role of the DCS in the regulation of ion transport in the intestine is important for considering the host defense mechanism and the energy metabolism as well as for elucidation of metabolic pathogenesis and the development of new therapeutics.

## Figures and Tables

**Table 1:** Overview of enteroendocrine cells along the gastrointestinal tract.

Cell Types	Gut Hormones	Gastric Body	Gastric Antrum	Small Intestine	Colon
EC	Serotonin	+	+	+	+
D	Somatostatin	+	+	+	+
ECL	Unknown	+			
A	Glucagon	+ (in fetus)			
X = A-like	Ghrelin	+			
G	Gastrin		+	+	
M = I	CCK			+	
S	Secretin			+	
K	GIP			+	
Mo	Motilin			+	
N	Neurotensin				
L	GLP/PYY			+	+

Abbreviations: CCK, cholecystokinin; EC, enterochromafin; ECL, enterochromaffin-like; GIP, glucose-dependent insulinotropic polypeptide; GLP-1, glucagon-like peptide 1 and 2; Mo, motilin; PYY, peptide YY.

## Abbreviations

ACh	acetylcholine
CGRP	calcitonin gene-related peptide
4-CHCA	a-cyano-4-hydroxycinnamic acid
eNOS	endothelial nitric oxide synthase
FFA2	free fatty acid receptor 2
FFA3	free fatty acid receptor s
5-HT	5-hydroxytriptamine
GLP-1	glucagon-like peptide 1
MQC	*N*-[2-methylphenyl]-[4-furan-3-yl]-2-methyl-5-oxo-1,4,5,6,7,8-hexahydroquinoline-3-carboxamide
NO	nitric oxide
PA1	phenylacetamide 1
PGE_2_	prostaglandin E_2_
PTX	pertussis toxin
SCFA	short-chain fatty acid
TTX	tetrodotoxin
VIP	vasoactive intestinal polypeptide
